# Colorectal Cancer: 35 Cases in Asbestos-Exposed Workers

**DOI:** 10.3390/healthcare11233077

**Published:** 2023-11-30

**Authors:** Antonietta Porzio, Alessandro Feola, Giuseppe Parisi, Angelo Lauro, Carlo Pietro Campobasso

**Affiliations:** 1 Department of Experimental Medicine, University of Campania “Luigi Vanvitelli”, Via Luciano Armanni 5, 80138 Naples, Italy; antonietta.porzio@unicampania.it (A.P.); giuseppe.parisi3@studenti.unicampania.it (G.P.); carlopietro.campobasso@unicampania.it (C.P.C.); 2 Department of Mental and Physical Health and Preventive Medicine, University of Campania “Luigi Vanvitelli”, Via Luciano Armanni 5, 80138 Naples, Italy; 3 Regional Directorate Campania, National Institute for Insurance against Accidents at Work (INAIL), 80143 Napoli, Italy; a.lauro@inail.it

**Keywords:** asbestos, colon cancer, asbestos exposure, occupational cancer

## Abstract

Background: Asbestos is considered one of the major global work-related carcinogens. Some studies suggest a potential causal relationship between asbestos exposure and colorectal cancer (CRC). However, the role of asbestos in CRC carcinogenesis is still controversial. Methods: 35 claims of occupational CRC among asbestos-exposed workers were reviewed. All claims were rejected by the Italian National Institute for Insurance against Accidents (INAIL) due to the “lack of scientific evidence in the causality” between asbestos exposure and CRC; causality was finally assessed in civil trials. All cases were categorized by age, gender, industry type, task, exposure and latency periods, anatomical location, and histopathological characteristics of CRC and concomitant respiratory diseases. Results: Most workers were males aged 60 years or more and employed in occupational activities with extensive use of asbestos for over 20 years. In 31 out of 35 cases, CRC was diagnosed over 39 years after employment. Right-sided colic adenocarcinoma was diagnosed in nine cases; rectum was involved in eight cases. Respiratory comorbidities were observed in 22 workers. Conclusions: Our study provides some interesting points in the assessment of the causal relationship between asbestos exposure and CRC.

## 1. Introduction

About 231,000 cases of asbestos-related occupational diseases are globally documented every year. [1]. According to the World Health Organization (WHO), more than 90,000 asbestos-related deaths are accounted for [2].

Italy was one of the major asbestos producers in Europe until 1992, when its extraction, commercialization, and production were banned [3]. For this reason, Italy is the country with the highest malignant mesothelioma death rates [4,5]. From 2017 to 2021, approximately 1400 asbestos-related diseases per year have been diagnosed in Italy [6], and from 2010 to 2016, asbestos-related deaths were about 4000 per year [7].

However, mortality rates are only related to patients suffering from mesothelioma, lung and larynx cancers, ovarian cancer, and asbestosis. Based on the carcinogenetic evidence assessed by the International Agency for Research on Cancer—I.A.R.C., these conditions are associated *with sufficient evidence in humans* (Group 1) with asbestos exposure [8], whereas other cancers and non-malignant pleural diseases are not considered. In this regard, the 2012 IARC Monographs on asbestos established a positive association between asbestos exposure and stomach, pharynx, and colorectal cancer (CRC), but *with limited evidence in humans* (Group 2A). For CRC specifically, the IARC Working Group was divided as to whether the evidence was *sufficient* to classify the association between CRC and asbestos in Group 1.

Although CRC has not generally been considered an occupational disease caused by asbestos, increased risks of CRC have been reported for workers exposed to chemical compounds and asbestos in several industrial branches [9,10].

In 1964, an American cohort study on asbestos-exposed populations concluded that there was an etiologic relationship between industrial asbestos exposure and bowel cancer [11]. A significant positive association between cumulative exposure to asbestos and the incidence of colon cancer was also observed in a large prospective French cohort of 14,515 men, including 181 colon cancer and 62 rectal cancer cases [12].

Other cohort studies suggest that asbestos exposure can increase the risk of developing CRC and dying from it [13,14]. A high cumulative exposure > 40 fiber-years/mL showed a positive trend with an increase in CRC mortality rates [15]. Further studies [16] reported a significant association between duration of exposed work and CRC among workers with asbestos exposure > 20 years. Several meta-analyses [17,18,19] also reported an elevated standardized mortality ratio for CRC in asbestos-exposed workers. Focusing on the anatomical location of CRC in asbestos-cement workers, an increased incidence of malignancy in the right part of the colon but not in the left side was observed [20]. A 2008 meta-analysis, instead, reported no association between asbestos exposure and rectal cancer [21]. A latency period of more than 39 years, from the beginning of occupational exposure to CRC diagnosis, was found to increase the risk of CRC [22].

Other studies [23,24] found no relationship between asbestos exposure and CRC. Therefore, the role of asbestos in CRC carcinogenesis is controversial [25].

In Italy, the public system of safety for workers is managed by the Italian National Institute for Insurance against Accidents at Work (INAIL) [26]. INAIL is the national authority in charge of the compensation for workers affected by occupational diseases.

Based on the IARC “*List of classifications by cancer sites with sufficient evidence in humans*” [8], INAIL recognizes the occupational origin of CRC in X- and Gamma-radiation-exposed workers but not for laborers with asbestos exposure. However, the claims submitted to the Italian Civil Judicial Authority by the insured asbestos-exposed workers with CRC or their relatives are increasing. According to INAIL data [6], 265 occupational CRC cases were claimed from 2017 to 2021, of which only 14 were compensated by INAIL; an asbestos exposure was recognized for all of them. Four patients died from cancer evolution; therefore, compensation was paid to the worker’s relatives. 

A series of 35 claims promoted by workers with a CRC diagnosis and occupational exposure to asbestos in Campania are reported. Campania is the third most-populous Italian region, located on the southern west coast, with a population of around 5,800,000 people. It is also the third-most industrialized region in Southern Italy, with the highest number of asbestos-related diseases recognized by INAIL [27]. In 2019, 42 claims for occupational CRC due to asbestos exposure were recorded in Italy, among which 15 (35.7%) were in Campania [6].

The aim of the study is the detection and analysis of similarities and differences among the CRC cases assessed as occupational diseases in order to improve knowledge on the controversial role of asbestos in the carcinogenesis of CRC.

## 2. Materials and Methods

A series of claims submitted to the Campania Civil Judicial Authority for recognition of the occupational origin of the CRC have been reviewed. Occupational exposure to asbestos was recognized by INAIL in 35 cases that were therefore included in the study.

Despite being recognized as asbestos-exposed workers by INAIL, the claims for occupational cancer were rejected due to the “lack of scientific evidence in the causality” between asbestos exposure and CRC. However, the causal relationship between asbestos exposure and CRC was later assessed during the civil trials. 

Patients were categorized by gender, age, type of industry tasks, risk factors, histopathological characteristics of CRC (staging, grades, and metastatic pattern), anatomical location (right colon, left colon, and rectum), and pleural and pulmonary comorbidities.

According to Baran et al. [28], the CRC anatomical location was assessed as follows: the right-sided colon cancer, from the cecum to the not-included splenic flexure; the left-sided colon cancer, from the splenic flexure to the rectum; and the rectum cancer. The rationale for collecting data on the location of the cancer comes from the results of previous studies on occupational CRC in workers of some industrial branches [21,22], which reported elevated risks of right-sided colon cancer.

The malignancy staging was assessed according to the TNM classification system provided by the 8th Edition of the American Joint Committee on Cancer (AJCC) Cancer Staging Manual [29]. Details are provided in Table 1.

The determination of tumor grade was assessed microscopically according to the 2-tiered grading system, which combines well and moderately differentiated to low grade (50% gland formation) and defines poorly differentiated as high grade (<50% gland formation) [30].

Additional data, such as the duration of exposure to asbestos fibers (the time period from the beginning of the occupational exposure to the end of the work at risk) and the latency period (defined as the time period from the beginning of occupational exposure to the CRC diagnosis), were collected. In the case records, the assessment of airborne asbestos fiber concentration by the Technical Advisory Department for Risk Assessment and Prevention was not included. So, individual exposures over a lifetime work history were not available.

## 3. Results

As shown in Table 2, patients were categorized according to gender, age, type of industry and occupational activity, duration of asbestos exposure and latency period, anatomical location and CRC stage, respiratory comorbidities, risk factors for CRC, and time of death, before or after the claim.

Most of the asbestos-exposed workers were males older than 60 years of age. Only two women over 75 were accounted for in the study sample. The average age of the study sample was 70.14 years. The youngest worker was 57 years old, while the oldest one was 90 years old.

A total of 33 out of 35 workers were exposed to asbestos fibers for over 15 years, while in two cases the occupational exposure was no less than 8–10 years. The observed latency period ranged from 33 to 66 years.

Most workers were employed in occupational activities with a large use of asbestos, except one laborer in the paper industry. Table 3 shows the distribution of occupational activities among the study sample.

A total of 22 out of 35 workers (62.8%) were employed in the steel industry, including steel and synthetic fiber manufacturing but also processing asbestos-containing products. Three workers were employed in shipbuilding companies where the main occupational activities were represented by the installation, repair, or removal of asbestos insulation [31]. A total of 9 out of 35 workers (25.7%) were employed in the metalworking industry, producing supplies for road and railway infrastructures and for industrial products and equipment. The only two females were both employed as maintenance workers for a steel company. They died after a few months from the CRC diagnosis, and the claims for the recognition of the occupational cancer were submitted by a family member.

Table 4 shows the main characteristics of the study sample, categorized by gender, age, anatomical location of CRC, risk factors, and respiratory comorbidities. A total of 27 out of 35 workers were diagnosed with colon adenocarcinoma (COAD). Therefore, the colon was the main anatomical location of the tumor. In 14 out of the 35 cases, the anatomical location of colon cancer was unspecified. Rectum was involved in eight laborers (22.8%); right-sided and left-sided colon cancer was assessed in nine (25.7%) and three workers (8.5%), respectively. Only one case of multifocal cancer was observed.

All nine workers with right-sided COAD were males, with an average age of 66 years. The occupational asbestos exposure was over 15 years (range 15–31 years), with a mean latency period of 44 years (range 33–49 years). No CRC-related deaths were observed among the right-sided COAD group before the submission of the claim, except for a 60-year-old worker with high-grade CRC and pulmonary fibrosis. Concomitant lung diseases were observed in eight out of nine workers with right-sided COAD, among which asbestosis was also diagnosed in two patients (cases #4 and #7).

**Figure 1:**
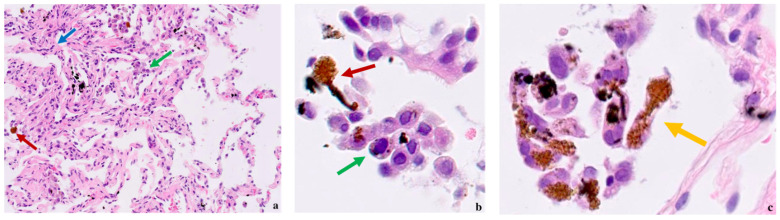
Lung histology of case #4 with ascending COAD and asbestosis. (**a**) Diffuse interstitial fibrosis (blue arrow) with macrophages (green arrow) and asbestos bodies (red arrow) in the alveolar spaces (HE-20×); (**b**) several alveolar macrophages (green arrow) with an asbestos body (red arrow) in the alveolar space (HE-80×). (**c**) A dumbbell-shaped ferruginous body with visible asbestos fibers inside (yellow arrow) (HE-100×).

**Figure 2:**
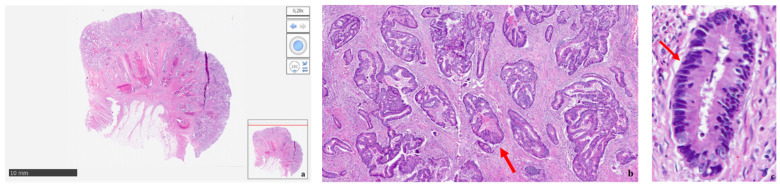
Colon histology of case #4 with ascending COAD and asbestosis. (**a**) Section overview of ascending colon with infiltrative growth pattern adenocarcinoma. (**b**) Well-differentiated adenocarcinoma with gland formation (red arrow) (HE-20×); (**c**) differentiated adenocarcinoma with well-formed glands and basally oriented nuclei (red arrow) (HE-30×).

In this regard, case #4, which is about a 67-year-old male diagnosed with locally advanced ascending colon cancer, is quite representative. He had been working since 1971 as a mechanic and overhead crane operator for a steel company for a total of 18 years. COAD was diagnosed 47 years after being employed. No risk factors for CRC other than age were found. Asbestosis was detected via high-resolution computed tomography (HRCT). Asbestos-related histological findings were observed in biopsy samples of the lung and colon. In lung sections, asbestos bodies in the alveolar spaces with diffuse interstitial fibrosis and macrophages were found (Figure 1a–c), while sections of the ascending colon showed an infiltrative growth-pattern adenocarcinoma with basal nuclei (Figure 2a–c). No asbestos fibers or ferruginous bodies were identified in the colon sections.

Respiratory comorbidities were observed in 22 out of 35 workers (62.8%), among which five cases of asbestosis (22.7%) had a typical inflammatory and fibrotic response involving the lower lobes. In two cases (cases #7 and #15), the upper lobes were involved with honeycomb aspects due to the advanced stage of asbestosis, and in the other two cases (cases #16 and #22), pleural parietal plaques were also observed, as frequently detected in asbestos-exposed workers [32,33,34].

In four workers interstitial pneumonia was observed with different radiological patterns as the reticulonodular, pseudonodular, and ground glass opacities (GGO). Other respiratory comorbidities were represented by lung metastases from CRC in three workers (cases #5, #14, and #24) and two nodules of undefined origin (cases #10 and #26). Table 5 shows the distribution of concomitant respiratory diseases among the 22 workers.

In 31 out of 35 cases, the cancer staging was defined according to the TNM classification system [29]. In only five cases, the cancer was in the bowel submucosa (T1); in others, the tumor had spread into the muscularis propria (T2; nine cases) or into the subserosa (T3; sixteen cases). One case of locally advanced neoplasia penetrating the surface of the visceral peritoneum was detected. Regional lymph nodes were involved in 48.5% (17 cases of 35), while the cancer had spread to other parts of the body with liver metastasis in 7 workers (cases #2, #6, #16, #21, #26, #32, and #33) and lung metastasis in 3 cases. According to the two-tiered system, in 14 out of 35 cases (40%), a high-grade cancer with poorly differentiated cells was diagnosed. In seven cases, cancer cells were well or moderately differentiated, with >50% gland formation. In 14 cases, microscopical findings were not available to define the CRC grade.

Data on CRC-related risk factors other than age were not always available. However, they were found in six cases in total. Smoking was the most common and reported risk factor in four cases, followed by alcohol use familial adenomatous polyposis (F.A.P.) in one patient each.

A death certificate was available only for 16 out of the 35 dead from CRC evolution or from therapy side-effects before the submission of the claims. In these cases, among the two females in the sample study, claims were submitted by the worker’s relatives. A total of 19 claims of occupational disease were submitted directly by the laborers when they were still alive, but it is not possible to assess the cause of death or if they died soon after the end of the trial or later. No information on the subsequent clinical course of these workers is available.

## 4. Discussion

CRC is one of the most frequent tumors around the world. In 2020, more than 1.9 million new cases of CRC and over 930,000 CRC-related deaths were reported worldwide [35]. CRC is the third-leading cause of cancer in Italy [36]. According to the Italian Association of Clinical Oncology (AIOM), CRC has been diagnosed in over 500,000 Italian citizens (280,277 males and 233,245 females), and in 2021, approximately 21,700 patients died from CRC [37]. Due to the slowdown in cancer screening, new CRC diagnoses dropped during the COVID-19 pandemic. However, in 2022, 48,100 new cases were reported worldwide, defining a “real colorectal cancer epidemic” [37].

The etiology of CRC has not been fully explained, and the immediate causes are still unknown [38]. Gene mutations, epigenetic alterations, and local inflammatory changes can certainly play a carcinogenetic role in CRC [39]. Some risk factors are strongly linked to an increased risk of developing the disease, among which age is considered one of the majors [38]. The risk of developing CRC increases as people become older, since the majority of CRC occurs in people older than 50 years of age [40]. For colon cancer, the average age at the time of diagnosis is 68 years for males and 72 years for females. For rectal cancer, the average age is 63 years for both males and females [40]. 

Other exogenous risk factors for CRC include dietary and lifestyle [41], smoking [42], heavy alcohol assumption (daily average > 3 drinks) [43], obesity (body mass index ≥ 30 kg/m^2^) [44], and excessive red meat intake (100 g/day) [45]. 

The scientific community agrees on the correlation between demographic, environmental, and lifestyle factors and the risk of developing CRC. On the contrary, the carcinogenetic role of asbestos remains controversial, explaining why INAIL commonly rejects the claims of asbestos-exposed workers with CRC, as reported in our study.

There is no agreement on the pathway followed by asbestos fibers to get to the digestive tract and its role in the development of CRC. According to I.A.R.C. monograph 100 C (updated in 2012), there are studies with discrepant results on the occurrence of gastrointestinal tract cancer following asbestos ingestion [8]. Asbestos fibers have been found in drinking water as the result of the deterioration of asbestos-containing materials like asbestos cement pipes and water tanks still present in water supply systems [46]. The data available in the scientific literature about the toxicokinetics and metabolism of asbestos following ingestion have been reviewed in a 2021 WHO report on the “*Guidelines for drinking-water quality*” [47]. Asbestos bodies were also found via light and electron microscopy in colon tissue from patients with occupational asbestos exposure [48,49,50], as well as in internal organs other than the lungs. Auerbach et al. [51] examined specimens of different tissues collected at autopsy from 37 occupational victims with asbestosis, mesothelioma, pleural plaques, and lung cancer. Asbestos bodies were observed in the liver, mediastinal lymph nodes, pancreas, omentum, duodenum, stomach, and colon. A recent Italian study [52] reported four cases of CRC in asbestos-exposed patients developing synchronous or metachronous mesothelioma. Asbestos bodies were histologically identified in cytoblocks of ascitic fluid. However, the role of asbestos fibers ingested with drinking water and their pathway to the gastrointestinal tract wall in order to cause adverse carcinogenic effects is still under debate [53,54,55].

Therefore, CRC, like other tumors or chronic degenerative diseases, is not commonly recognized as an occupational disease [10].

However, elevated risks of CRC have been reported among workers in some industrial branches, such as the textile industry [56,57], the car industry [58,59,60], the beverage industry [61], and also among laborers exposed to asbestos [8,48,62,63], dioxin [64], wood dust [65], organic solvents [66,67,68], and metal-working fluids [69]. In fact, one of the main topics to be assessed in the causal relationship between asbestos and CRC is the presence of carcinogens other than asbestos in the occupational environment [70]. An increased risk of CRC has also been recognized for workers employed in industries with a wide use of chemical compounds like leather, basic metals, plastic, and rubber manufacturing, besides laborers in the sector of repair and installation of machinery exposed to asbestos [10]. IARC also suggested a positive relationship *with limited evidence in humans* between CRC and other substances such as nickel and nickel compounds, erionite, and wood dusts [8].

In our study sample, most of the asbestos-exposed workers were old males over 60 years of age, except for two females over 75 years of age. The youngest worker was 57 years old, but the mean age of the sample study was 70.14 years.

All claims reviewed dealt with workers employed in occupational activities with a large use of asbestos, like steel and metalworking companies and shipbuilding industries. Three workers were employed in a metalworking factory producing supplies for road and railway infrastructures and for industrial products and equipment. In this regard, rail transport equipment mechanics and repairers exposed to asbestos have been associated with a high risk of CRC [71]. An excess risk of developing CRC has also been reported among iron and steel workers [72]. A total of 22 out of 35 cases, including the two women in our sample study, worked for a steel company as maintenance laborers. Most of the workers were employed in an asbestos-cement plant located in Naples with high asbestos environmental pollution. A previous study [73] reported an airborne asbestos fiber concentration ranging from 0.030 to 1.033 ff/cc from environmental samplings collected in different areas of this plant. The Italian National Institute of Health, considering the cumulative exposure index (CEI) by industrial sector, observed a CEI > 620 f/mL for workers in the asbestos cement industry; for shipyard and railway rolling stock, the maximum exposure indices were, respectively, equal to 195 f/mL and 146.6 f/mL [74]. For our sample, the technical evaluation of the airborne asbestos fibers’ concentration by the regional Technical Advisory Department for Risk Assessment and Prevention was not available. So, it was not possible to estimate individual cumulative doses.

All the laborers were hired between 1955 and 1976 and stopped working before or a few years after the asbestos ban was approved in Italy in 1992. INAIL recognized to all these workers the risk of asbestos exposure but not the causal relationship with CRC. 

According to the 2019 INAIL report [75], most of the technopathies claimed by the Italian workers are coming from the Industry and Services. Among these technopathies, the asbestos-related diseases recognized by INAIL between 2017 and 2021 were 7145 [27]. Most of the workers (26 out of 35 cases) were exposed to asbestos fibers for over 20 years. According to previous cohort studies, a large exposure like this increased the risk of CRC [16], particularly applying to the nine male workers with right-sided colon cancer exposed to asbestos fibers for over 15 years. In 31 out of 35 cases, the diagnosis of CRC was assessed approximately over 39 years after the beginning of their occupational exposure. This is in agreement with other studies [15,61] that observed a temporal relationship between asbestos exposure and CRC development, with a minimum latency period of 15–20 years. On latency, other studies considered a period of exposure preceding malignancy by 40 years to detect an elevated risk of CRC [22,76].

In the study sample, the colon represented the main anatomical location of the tumor in 27 out of 35 workers. Although COAD was not sided in 14 out of 27 cases, the right colon was involved in nine workers, the left colon in three laborers, and a single case of multifocal cancer was observed. The rectum was involved in eight workers.

Furthermore, in 22 out of 35 workers, concomitant asbestos-related lung diseases were also detected. Asbestosis was found in five cases, followed by interstitial pneumonia with classical imaging, lung metastases from CRC, pleural plaques, and nodules. In this regard, it is worth mentioning that in previous studies, the highest risk of CRC has been observed in patients with asbestos-induced pleural plaques [77]. The relative risk of CRC also increased with the worsening asbestosis. An increased mortality rate for CRC among Italian females with asbestosis was also reported [78], but none of the two female workers in our sample study showed asbestosis. All five laborers with concomitant asbestosis received an INAIL benefit for the occupational origin of the asbestosis.

Based on recorded anamnestic and clinical data, CRC-related risk factors were detected only in six patients. In our study sample, smoking was the most common exogenous factor; one worker reported a history of alcohol consumption, and in one case, a classic familial adenomatous polyposis was genetically diagnosed.

The authors are aware of the limitations of this study. Our sample size is poor, with only 35 cases. Also, it was not possible to determine the individual cumulative exposure to asbestos, understood as the product of the concentration of asbestos fibers (per milliliter of air measured) and the duration of exposure in years [79]. No information on the cumulative dose of asbestos was provided by individual and/or environmental health surveillance.

The death certificate was available only for 16 out of 35 workers dead from CRC evolution before the submission of the claims. Information dealing with the subsequent clinical course of the CRC was not available in 19 cases. Furthermore, an increase in CRC mortality rate has been observed in heavily asbestos-exposed cohorts [15,80]; however, further research is needed to compare this evidence to our sample study.

## 5. Conclusions

There is still no general agreement on the carcinogenetic role of asbestos in CRC cases. Acknowledging the small number of the sample, this study provides some interesting points in the assessment of Due to the limitations of the sample study, our case series does not provide enough evidence of the causal relationship between asbestos exposure and CRC. An improvement in research on CRC pathogenesis and asbestos-related diseases is needed to determine the possible carcinogenetic role of asbestos in CRC cases.

Results are consistent with data provided by previous studies, showing several similarities in latency period, time of exposure, and anatomical site of malignancy. Six patients had a history of non-occupational personal risk factors (smoking, F.A.P., and alcohol use), but the possibility of an increased risk of CRC from asbestos exposure cannot be excluded. 

Despite not providing enough evidence on the causal relationship between asbestos exposure and CRC, our case series represents a starting point for further studies.

Workers exposed to asbestos should be made aware of the possibility of CRC and should undergo monitoring and medical examinations. A specific screening program for workers with CRC previously exposed to asbestos and other chemical compounds in specific industrial branches could also be useful to collect more data and support the final evaluation of the occupational origin.

Experts should be cautious and collect whenever possible all useful information about the clinical and occupational history of the worker, focusing on the type of occupational activities, cumulative exposure to asbestos or other chemical compounds, latency period, lifestyle, anatomical site, staging, and histotype of the tumor.

A detailed personal and professional anamnesis including lifestyle, environmental, and occupational risk factors could be of help in the determination of occupational CRC in asbestos-exposed workers.

A specific screening program for workers with CRC previously exposed to asbestos and other chemical compounds in specific industrial branches could also be useful to collect more data and support the final evaluation of the occupational origin.

The global burden of the CRC is projected to increase to 3.2 million new cases and 1.6 million deaths by 2040 [78]. More prevention policies should be established. Standardized protocols for collecting individual and occupational data could be adopted in the diagnostic evaluation of occupational CRC disease. Among the others, useful data are represented by the type of occupational activities, duration of exposure, cumulative exposure to asbestos or other chemical compounds, latency period, lifestyle, genetic diseases, stage and location of the tumor (including metastases and histotype), and results of histology and immunohistochemistry on biopsy samples or tissue samples collected at autopsy.

The right of all workers to be safe and healthy at work needs to be protected, as well as the right to grant compensation to workers affected by any occupational disease.

From a forensic perspective, the assessment of the potential role of asbestos in CRC carcinogenesis requires further research, involving both histopathological and immunochemical analysis of the samples collected at autopsies of asbestos-exposed workers deceased for CRC.

## Figures and Tables

**Table 1:** TNM classification system for CRC according to AJCC.

Primary Tumor (pT)
**TX:** primary tumor cannot be assessed **T0:** no evidence of primary tumor **Tis:** carcinoma in situ, intramucosal carcinoma **T1:** tumor invades submucosa **T2:** tumor invades muscularis propria **T3:** tumor invades through the muscularis propria into the pericolorectal tissues **T4a:** tumor invades through the visceral peritoneum **T4b:** tumor directly invades or adheres to other adjacent organs or structures
**Regional lymph nodes (N)**
**NX:** regional lymph nodes cannot be assessed **N0:** no regional lymph node metastasis **N1:** metastasis in 1–3 regional lymph nodes *N1a*: metastasis in 1 regional lymph node *N1b:* metastasis in 2–3 regional lymph nodes *N1c:* tumor deposits in the subserosa, mesentery or non-peritonealized pericolic or perirectal/mesorectal tissues **N2:** metastasis in 4 or more regional lymph nodes *N2a:* metastasis in 4–6 regional lymph nodes *N2b*: metastasis in 7 or more regional lymph nodes
**Distant metastasis (M)**
**M0:** no distant metastasis by imaging; no evidence of tumor in other sites or organs **M1:** distant metastasis *M1a:* metastasis confined to 1 organ or site without peritoneal metastasis *M1b*: metastasis to 2 or more sites or organs is identified without peritoneal metastasis *M1c:* metastasis to the peritoneal surface is identified alone or with other site or organ metastases

**Table 2:** The items collected from 35 claims of asbestos-exposed workers.

*n*	Gender	Age (Years)	Task	Asbestos Exposure (Years)	Latency Period (Years)	CRC Site and Grade	pTNM	Lung Diseases	Other Risk Factors	Death (Before/After)
1	M	74	Mechanical operator, gas operator in coking department	20	47	R-COAD Low grade	pT1 N0 M0	Interstitial disease	-	After
2	M	68	Mechanical operator, oven worker in steel mill	29	49	R-COAD High grade	pT3 N2b M1	Interstitial disease	-	After
3	M	67	Mechanical operator	17	44	R-COAD High grade	pT4a N1a M0	Pulmonary fibrosis	-	After
4	M	67	Mechanical operator, overhead crane operator	20	47	R-COAD High grade	pT3 N0 M0	Asbestosis	-	After
5	M	68	Reamer	31	49	R-COAD High grade	pT3 N1a M1	CRC metastasis	Smoking	After
6	M	60	Logistic manager, carpenter	22	39	R-COAD High grade	pT2 N1a M1	Pulmonary fibrosis	-	Before
7	M	72	Mechanical operator, repairer	26	48	R-COAD High grade	pT1 N0 M0	Asbestosis	-	After
8	M	61	Programmer	15	40	R-COAD Low grade	pT3 N0 M0	-	-	After
9	M	58	Assistant logistic manager	20	33	R-COAD High grade	pT3 N0 M0	Emphysema-COPD	-	After
10	M	69	Pipefitter	22	45	L-COAD High grade	pT3 N1 M0	Nodule	Smoking	After
11	M	76	Mechanical operator	28	53	L-COAD High grade	pT3 N2 M0	-	Smoking	After
12	M	69	Mechanical operator	18	44	L-COAD Low grade	pT1 N0 M0	Pseudo-nodule	-	After
13	M	75	Electrician	31	55	Multifocal COAD High grade	pT1 N0 M0	Interstitial disease	-	Before
14	M	73	Carpenter, welder	21	39	ROAD High grade	pT3a N1a M1	CRC metastases	-	Before
15	M	65	Mechanical operator	30	48	ROAD High grade	pT3 N1 M0	Asbestosis	Alcohol use	Before
16	M	57	Turner	22	34	ROAD High grade	pT3 N1 M1	Pleural plaques		Before
17	M	74	Bricklayer	26	50	ROAD High grade	pT2 N1 M0	-	-	After
18	M	68	Laborer, transport worker	8	41	ROAD Low grade	pT2 N0 M0	COPD	Smoking	After
19	M	75	Carpenter	21	41	ROAD Low grade	pT3 N0 M0	Interstitial disease	F.A.P.	Before
20	M	71	Carpenter	30	52	ROAD Low grade	pT2 N1 M0	COPD	-	After
21	M	79	Maintenance worker	29	66	ROAD Low grade	pT3 N1 M1	Asbestosis	-	After
22	M	90	Operator in insulation department	17	66	Unspecific site COAD	pT2 N0 M0	Pleural plaques	-	After
23	M	68	Carpenter	10	36	Unspecific site COAD	pT1 N0 M0	-	-	After
24	M	78	Operator in coking department	16	44	Unspecific site COAD	pT2 N0 M0	CRC metastases	-	Before
25	M	77	Mechanical operator	22	49	Unspecific site COAD	pT3 N0 M0	Asbestosis	-	After
26	M	72	Mechanical operator	19	45	Unspecific site COAD	pT3 N1b M1	Nodule	-	Before
27	M	74	Maintenance worker	19	37	Unspecific site COAD	pT3 N1 M0	-	-	Before
28	M	63	Mechanical operator	21	41	Unspecific site COAD	-	-	-	Before
29	M	62	Electrician	26	49	Unspecific site COAD	-	-	-	Before
30	M	75	Mechanical operator	23	40	Unspecific site COAD	pT2 N1 M0	-	-	Before
31	M	68	Maintenance worker	20	45	Unspecific site COAD	-	-	-	Before
32	F	77	Maintenance worker	27	51	Unspecific site COAD	pT2 N1 M1	-	-	Before
33	F	83	Maintenance worker	22	39	Unspecific site COAD	pT3 N1 M1	-	-	Before
34	M	57	Mechanical operator	23	43	Unspecific site COAD	-	-	-	Before
35	M	65	Mechanical operator	28	41	Unspecific site COAD	pT2 N0 M0	-	-	After

COAD = colon adenocarcinoma; R-COAD = right-sided colon adenocarcinoma; L-COAD = left-sided colon adenocarcinoma; and ROAD = rectal adenocarcinoma.

**Table 3:** Distribution of occupational activities in the sample study.

Industry Type	Cases	%
Shipbuilding industry	**3**	**8.57**
Paper industry	**1**	**2.86**
Metalworking industry	**9**	**25.71**
Production of industrial goods and machinery	6	17.14
Supplies for road and railway infrastructures	3	8.57
Steel industry	**22**	**62.86**
Asbestos processing	1	2.86
Steel manufacturing	13	37.14
Synthetic fibers production	8	22.86
**TOTAL**	**35**	**100**

**Table 4:** Summary of the sample study.

Characteristics of Patients	Cases	%
Age group (years)		
51–60	4	11.43
61–70	14	40.00
71–80	15	42.86
Over 80	2	5.71
Gender		
Male	33	94.29
Female	2	6.06
Site		
Right-sided colon	9	25.71
Left-sided colon	3	8.57
Unspecified colon site	14	40.00
Multifocal	1	2.86
Rectum	8	22.86
Respiratory comorbidity		
Present	22	62.86
Absent	5	14.28
**Data not available**	**8**	**22.86**
Risk factors		
Present	6	17.14
Smoking	4	11.42
F.A.P.	1	2.86
Alcohol use	1	2.86
Absent	21	60.00
**Data not available**	**8**	**22.86**
**TOTAL**	**35**	**100**

**Table 5:** Cases with concomitant respiratory disease.

Respiratory Comorbidities	Cases	%
Interstitial diseases	**4**	**18.18**
Reticulo-nodular pattern	2	9.08
Pseudo-nodular pattern	1	4.55
Ground Glass Opacities	1	4.55
Asbestosis	**5**	**22.73**
Pleural plaques	**2**	**9.09**
Nodules	**2**	**9.09**
Metastases	**3**	**13.64**
Non-specific lung disease	**6**	**27.27**
**TOTAL**	**22**	**100**

## Data Availability

The data presented in this study are available on request from the corresponding author.
